# Incidence and Risk of Anti-Resorptive Agent-Related Osteonecrosis of the Jaw after Tooth Extraction: A Retrospective Study

**DOI:** 10.3390/healthcare10071332

**Published:** 2022-07-18

**Authors:** Rieko Shimizu, Shintaro Sukegawa, Yuka Sukegawa, Kazuaki Hasegawa, Sawako Ono, Tomoya Nakamura, Ai Fujimura, Ayaka Fujisawa, Keisuke Nakano, Kiyofumi Takabatake, Hotaka Kawai, Hitoshi Nagatsuka, Yoshihiko Furuki

**Affiliations:** 1 Department of Oral and Maxillofacial Surgery, Kagawa Prefectural Central Hospital, Takamatsu 760-8557, Japan; de421021@s.okayama-u.ac.jp (R.S.); yuka611225@gmail.com (Y.S.); de421040@s.okayama-u.ac.jp (K.H.); tmy.nakamura@s.okayama-u.ac.jp (T.N.); sugar.x.48@gmail.com (A.F.); ayafuji05@gmail.com (A.F.); furukiy@ma.pikara.ne.jp (Y.F.); 2 Department of Oral Pathology and Medicine, Graduate School of Medicine, Dentistry and Pharmaceutical Sciences, Okayama University, Okayama 700-8525, Japan; pir19btp@okayama-u.ac.jp (K.N.); gmd422094@s.okayama-u.ac.jp (K.T.); de18018@s.okayama-u.ac.jp (H.K.); jin@md.okayama-u.ac.jp (H.N.); 3 Department of Pathology, Kagawa Prefectural Central Hospital, Takamatsu 760-8557, Japan; de19008@s.okayama-u.ac.jp

**Keywords:** anti-resorptive agent-related osteonecrosis of the jaw, bisphosphonate, denosumab, retrospective study, risk factor, tooth extraction

## Abstract

Bone-modifying agents (BMA) such as bisphosphonates and denosumab are frequently used for the treatment of bone metastases, osteoporosis, and multiple myeloma. BMA may lead to anti-resorptive agent-related osteonecrosis of the jaw (ARONJ). This study aimed to clarify the risk factors for and probabilities of developing ARONJ after tooth extraction in patients undergoing BMA therapy. In this study, the records of 505 target sites of 302 patients undergoing BMA who presented with mandibular fractures at the Department of Oral and Maxillofacial Surgery, Kagawa Prefectural Central Hospital, from March 2014 to January 2022, were retrospectively analyzed for the onset of ARONJ after tooth extraction. The following variables were investigated as attributes: anatomy, health status, and dental treatment. The correlation coefficient was calculated for the success or failure of endodontic surgery for each variable, the odds ratio was calculated for the upper variable, and the factors related to the onset of ARONJ were identified. The incidence rate of ARONJ was found to be 3.2%. Hypoparathyroidism was an important factor associated with ARONJ development. Thus, systemic factors are more strongly related to the onset of ARONJ after tooth extraction than local factors.

## 1. Introduction

Bone-modifying agents (BMA) such as bisphosphonates (BP) and denosumab (DMAb) are used to prevent osteoporotic fractures, bone metastases, and bone-related events in multiple myeloma. However, since the first case of BP-related osteonecrosis of the jaw was described by Marx in 2003 [1], cases of anti-resorptive agent-related osteonecrosis of the jaw (ARONJ) have been commonly reported [2,3,4].

In Japan, a position paper was published in 2016 by six related academic societies (The Japanese Society for Bone and Mineral Research, Japan Osteoporosis Society, Japanese Society for Oral and Maxillofacial Radiology, The Japanese Society of Periodontology, Japanese Society of Oral and Maxillofacial Surgeons, and The Japanese Society for Oral Pathology), which identified several known risk factors for the onset of ARONJ [5]. Factors that influence the onset of ARONJ can be divided into local or systemic factors, including the duration of BP or DMAb administration, demographics and lifestyles, comorbidities, and co-therapeutic agents. The American Association of Oral and Maxillofacial Surgery (AAOMS) position paper states that tooth extraction is a common predisposing event [6]. Tooth extraction is the most common dental surgery and an important treatment that cannot be avoided in daily clinical practice. However, the importance and prevalence of ARONJ and its risk factors after tooth extraction remain unclear [5].

The purpose of this study was to clarify the risk factors and probability of developing ARONJ after tooth extraction in patients undergoing BMA.

## 2. Materials and Methods

### 2.1. Study Design

A retrospective clinical study was conducted on the incidence and risk factors of osteonecrosis of the jaw in patients who underwent tooth extraction at the Oral and Maxillofacial Surgery Department of Kagawa Prefectural Central Hospital between March 2014 and January 2022.

### 2.2. Ethics Statement

This study was approved by the Institutional Review Committee of Kagawa Prefectural Central Hospital (approval number 1101, approved on 25 April 2022). Informed consent was obtained from all patients in this study. All data were anonymized and analyzed.

### 2.3. Patients

The following criteria were used to select target patient cases: among patients taking BMAs, including BP and DMAb, an antibody-targeting receptor activator of nuclear factor kappa-b ligand (RANKL) therapy, those who had “hopeless” teeth, a good general condition, and a sufficient prognosis for life were included. All tooth extractions were performed under local anesthesia with the consent of the patient.

Exclusion criteria were patients who had consented to surgery other than tooth extraction, such as dental implant placement surgery and bone ridge resection, patients who could not be followed up for 1 month or more, and those who had a history or ongoing radiation treatment in the facial area.

According to the above criteria, 302 patients (505 tooth extraction sites) with BMAs were treated during the study period.

### 2.4. Surgical Procedure and Postoperative Management

All tooth extraction procedures were performed under local anesthesia, with or without intravenous sedation. A single surgical team of seven, under the guidance of two professional oral and maxillofacial surgeons (YF and SS), performed tooth extractions. In all cases, 4–0 absorbent silk was used for sutures, and the sutures were removed 10–14 days after surgery (Surgisorb 4–0; Nicho Co., Ltd., Tokyo, Japan.) The postoperative clinical management was performed in routine care. Patients received antibiotics (amoxicillin hydrate, 250 mg every 8 h, and in patients with penicillin allergies, clarithromycin 200 mg every 12 h) for 2 days postoperatively, as well as non-steroidal anti-inflammatory drugs (celecoxib 400 mg for early pain or 200 mg for the second and subsequent doses, or acetaminophen 500 mg).

### 2.5. Outcome Variables

The resulting variable was the incidence of ARONJ after tooth extraction. ARONJ was diagnosed according to the contents of the position paper [6] of the AAOMS summarized as follows: (a) current or previous treatment with anti-resorptive or anti-angiogenic agents, (b) exposed bone or bone that can be probed through an intraoral or extraoral fistula in the maxillofacial region that has persisted for more than 8 weeks, and (c) no history of radiation therapy to the jaw or obvious metastatic disease to the jaw.

### 2.6. Predictor Variables

The predictor variables for this study consisted of sets of exposures considered to be convincingly related to ARONJ and were classified as attribute, anatomical, health status, and dental treatment variables. Attribute variables included age and sex. Health status variables included body mass index (BMI), height, weight, alcohol consumption, smoking, anemia, diabetes, hypocalcemia, hemodialysis, hypoparathyroidism, hyperthyroidism, rheumatoid arthritis, steroid intake, molecular-targeted drugs, hormone therapy, chemotherapy, radiotherapy, malignant tumor, breast cancer, lung cancer, prostate cancer, colorectal cancer, and multimyeloma. Anemia was defined as hemoglobin levels <12 g/dL for women and <13 g/dL for men, according to the World Health Organization guidelines [7]. Diabetes was defined as >6.5% HbA1c, according to the National Glycohemoglobin Standardization Program [8]. Anatomical variables included tooth position (anterior, premolar, and molar), maxilla/mandible, and left/right. Drug variables included drug usage period and drug holidays. The drug usage period was based on 4 years, which is considered a risk factor for the onset of ONJ, and BMAs were divided into a group of 4 years or more and a group of less than 4 years.

### 2.7. Statistical Analysis

Data were recorded in an electronic database throughout the study using Microsoft Excel (Microsoft Inc., Redmond, WA, USA). For statistical analysis, the digital database used was JMP version 14.2.0 for Macintosh (SAS Institute Inc., Cary, NC, USA). Categorical variables are presented as numbers and percentages, while continuous variables are presented as mean and standard deviation (SD). For the two-group comparison, we used the Mann–Whitney U test for continuous variables and the chi-square test or Fisher’s exact test for categorical variables. Statistical significance was set at a two-tailed *p*-value < 0.05. Any association in bivariate analyses (*p* < 0.05) was included in a multiple logistic regression analysis, which was subsequently used to provide adjusted odds ratios (ORs) to control the simultaneous effects of multiple covariates. Statistical significance was set at *p* < 0.05.

The correlation coefficient is a measure of the strength of the linear relationship between two data points or a random variable. In the range of 0 to 1, the onset rate of ARONJ increases with the increasing index. Conversely, in the range of 0 to −1, as the index decreases, the likelihood of ARONJ occurrence decreases. Thus, the correlation coefficient chart was plotted according to the variable data based on the number of occurrences of ARONJ. Further, an algorithm for calculating statistical properties was implemented in Python (version 3.7.13) using NumPy (version 1.21.5) and the Pandas library (version 1.3.5).

## 3. Results

### 3.1. Comparison of Distribution of Predictive Variables Based on ONJ Occurrence after Tooth Extraction

Table 1 shows the results of the statistical evaluation using bivariate analysis and comparison of the distribution of each predictor variable based on the occurrence of ONJ after tooth extraction. Of the 505 sites, the incidence of ONJ after tooth extraction was 3.2% at 16 sites. Variables statistically related to ONJ onset after extraction (*p* < 0.05) included age (*p* = 0.004) for demographic variables, hypoparathyroidism (*p* <0.001), hormone therapy (*p* = 0.002), chemotherapy (*p* <0.001), radiotherapy (*p* <0.001), and breast cancer (*p* = 0.01) for health status variables.

**Figure 1:**
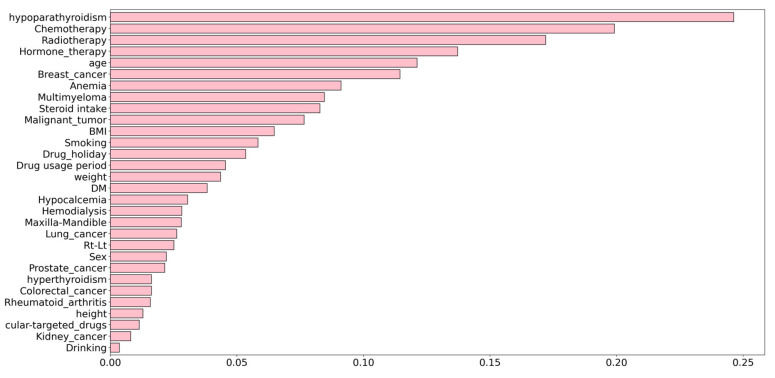
Correlation coefficients for the onset of osteonecrosis of the jaw (ONJ) after tooth extraction and predictive variables. The top five variables with the strongest correlation with ONJ onset were hypoparathyroidism, chemotherapy, radiotherapy, hormone therapy, and age.

When examining the correlation coefficient accounting for the prognosis of ONJ onset after tooth extraction, the top five factors were hypoparathyroidism, chemotherapy, radiotherapy, hormone therapy, and age (Figure 1). Among the items of the main predictors, the items that showed a significant difference on bivariate analysis and those with the highest correlation coefficient were the same.

### 3.2. Multivariate Logistic Regression Model Results of ONJ Onset after Tooth Extraction

For further statistical exploration, we used a multivariate logistic regression model for the occurrence of ONJ after tooth extraction, using significant variables in the bivariate analysis and variables with higher correlation coefficients. The selected items were hypoparathyroidism, chemotherapy, radiotherapy, hormone therapy, and age (Table 2).

In multivariate logistic regression analysis, hypoparathyroidism was a significant factor in the onset of ONJ. Chemotherapy showed a high OR for the extracted variables (OR = 3.617). It was also suggested that hypoparathyroidism is most involved in the onset of ONJ. However, it was difficult to properly calculate the OR because ONJ occurred in almost all patients with hypoparathyroidism.

## 4. Discussion

This retrospective study investigated the prevalence of and risk factors for ARONJ. The incidence of ARONJ in the present study was 3.2%, while the reported incidence of ARONJ is 17.8%, which is higher than the estimated value of 0.001–0.01% [9] reported by the International Task Force on ONJ. Our study also demonstrated that hypoparathyroidism was a strong risk factor for ARONJ after tooth extraction and that systemic factors are more strongly related to ARONJ onset after tooth extraction than local factors.

The risk of ARONJ onset in hypoparathyroidism has not yet been explored. Hypoparathyroidism is an endocrine disorder in which parathyroid hormone (PTH) production is abnormally low or absent [10]. It is associated with mild symptoms such as muscle cramps and malaise, and severe symptoms such as tetany, seizures, and cardiac dysrhythmia. PTH is also a regulator of bone structure and remodeling, and it was previously thought that in the absence of PTH in hypoparathyroidism, the incidence of fractures decreases due to increased bone mass and decreased remodeling. However, recent reports have shown that patients with hypoparathyroidism have increased cortical and trabecular width and decreased bone microstructure, including decreased cortical porosity and fractures due to decreased BMAs. This has been suggested to increase the risk of fracture [11,12]. BMAs are one of the main treatment options for osteoporosis and ARONJ is a side effect of BMA use. The mechanisms of ONJ development with BMA use include impaired bone remodeling, inflammation or infection, immune changes due to anticancer drugs and diabetes, and healing failure due to impaired angiogenesis. Since hypoparathyroidism causes a bone remodeling disorder, it is considered that the risk of ARONJ increases due to the synergistic effect with BMA administration [13]. In addition, PTH therapy is administered in hypoparathyroidism, and hormone therapy is suggested as a high risk for ARONJ in this study because of its correlation with hypoparathyroidism.

In addition, breast cancer had a high correlation with ARONJ; however, in patients with metastases, intravenous BPs or RANKL inhibitors were used, which reduced bone metabolism and turnover and resulted in ARONJ. The risk was therefore considered to be high in such cases. Moreover, radiation therapy to organs other than the jawbone is often administered to such cancer patients, and the risk factors are thus inevitably increased. Chemotherapy, such as BMA, suppresses bone metabolism and causes immune changes; hence, it is also considered a risk factor for ARONJ [14,15,16]. This study showed that breast cancer, radiation therapy, and chemotherapy factors were more strongly correlated with ARONJ onset than were other factors. However, they are not the absolute risk factors for ARONJ, and future studies examining the total dose of radiation therapy, type of chemotherapy, and accompanying changes in the patients’ general condition are warranted.

Regarding the BMA drug holiday and administration period, the AAOMS position paper (2014) advocates a 2-month drug holiday given the risk of ARONJ in those treated with BP for 4 years or more [6]. However, this notion is unsupported by scientific evidence. During the drug holiday, BP remains in the bone because it is incorporated into the calcified bone matrix, and bone clearance can range from weeks to years. Therefore, the drug holiday of BP is expected to have limited or no effect on bone turnover [15,17,18]. Meanwhile, nitrogen-containing BPs promote bacterial adhesion and biofilm formation, which can cause ARONJ by angiogenesis suppression and soft-tissue toxicity. Since infection of bone treated with BMA leads to the onset of ARONJ, it is important to avoid local infection by antibiotic administration and use of effective wound closure techniques, regardless of the drug suspension or administration period.

We used the correlation coefficient as a statistical tool to identify the risk factors responsible for the onset of ARONJ due to tooth extraction. Although most ranks of the correlation coefficient were similar to those of odds ratios obtained by multivariate analysis, some ranks were distinct. The correlation coefficient is an ordinal scale, and it is not possible to compare the strengths of causality. Therefore, it is necessary to identify risk factors using odds ratios by multivariate analysis. The advantage of calculating the correlation coefficient is that one can quickly and visually evaluate the correlation between two factors [19]. Our study is novel in that it is the first to identify risk factors for tooth extraction-induced ARONJ using correlation coefficients.

This retrospective study has two limitations. The first limitation is that the sample composition is biased. Since there are many referrals of patients with ARONJ to our department, the incidence of bone resorption inhibitor-related osteonecrosis of the jaw is higher than the value estimated by the International Task Force on Osteonecrosis of the Jaw. Therefore, future studies with a larger number of cases are necessary. Second, we could not confirm the treatment for comorbidities without treatment at our hospital. Therefore, confirmation of the treatment of comorbidities may be necessary for more accurate estimates in future studies. Despite the above limitations, to the best of our knowledge, multifactorial factors such as comorbidities and associated treatments were detected as risk factors for ARONJ in this study. In the future, for patients with comorbid diseases that are risk factors, active dental intervention should be performed on teeth that are the source of infection before BMA administration.

## 5. Conclusions

This retrospective study investigated the prevalence and risk factors for ARONJ after tooth extraction. The incidence of ARONJ was 3.2%, and we found that hypoparathyroidism was a strong risk factor for ARONJ after tooth extraction. Thus, systemic factors are more strongly related to the onset of ARONJ after tooth extraction than local factors.

## Figures and Tables

**Table 1:** Comparison of the distribution of predictive variables based on the onset of osteonecrosis of the jaw after tooth extraction.

		Healing	ONJ	*p* Value
Outcome (%)		489 (96.8)	16 (3.2)	
Demographic variables				
Age		75.82 ± 8.43	69.92 ±13.87	0.004
Sex				0.620
Male		70	3	
Female		419	13	
Health status variables				
BMI		22.01 ± 3.83	20.52 ± 3.84	0.057
Height		153.16 ± 8.86	154.03 ± 9.54	0.654
Weight		51.91 ± 11.82	48.91 ± 12.42	0.153
Drinking	Yes	371	12	0.9363
	No	118	4	
Smoking	Yes	422	12	0.201
	No	67	4	
Anemia	Yes	337	7	0.0524
	No	152	9	
Diabetes	Yes	422	15	0.390
	No	67	1	
Hypocalcemia	Yes	475	16	0.493
	No	14	0	
Hemodialysis	Yes	477	16	0.526
	No	12	0	
Hypoparathyroidism	Yes	489	15	<0.0001
	No	0	1	
Hyperthyroidism	Yes	485	16	0.716
	No	4	0	
Rheumatoid_arthritis	Yes	446	15	0.723
	No	43	1	
Steroid intake	Yes	396	10	0.067
	No	93	6	
Molecular-targeted_drugs	Yes	486	16	0.753
	No	3	0	
Hormone_therapy	Yes	472	13	0.002
	No	17	3	
Chemotherapy	Yes	444	9	<0.0001
	No	45	7	
Radiotherapy	Yes	484	14	0.000
	No	5	2	
Malignant_tumor	Yes	368	9	0.086
	No	121	7	
Breast_cancer	Yes	453	12	0.010
	No	36	4	
Lung_cancer	Yes	472	15	0.556
	No	17	1	
Prostate_cancer	Yes	482	16	0.630
	No	7	0	
Colorectal_cancer	Yes	485	16	0.716
	No	4	0	
Multimyeloma	Yes	484	15	0.058
	No	5	1	
Anatomic variables				
Tooth position				0.252
Anterior		153	2	
Premolar		209	8	
Molar		127	6	
Jaw				0.519
Maxilla		235	9	
Mandible		254	7	
Lt/Rt				0.572
Lt		249	7	
Rt		240	9	
Drug variables				
Drug usage period	Less than 4Y	339	13	0.3071
	More than 4Y	150	3	
Drug holiday	Yes	363	14	0.3799
	No	126	2	

**Table 2:** Multivariate logistic regression model results of the onset of osteonecrosis of the jaw after tooth extraction.

	Standard Error	OR	Lower 95% CI	Upper 95% CI	*p* Value
Hypoparathyroidism	22.373	-	-	-	<0.0001
Chemotherapy	1.275	3.617	0.987	13.258	0.055
Age	0.051	1.051	0.991	1.116	0.091
Radiotherapy	1.409	0.243	0.542	1.845	0.174
Hormone therapy	0.476	0.623	0.256	3.899	0.611

## Data Availability

The datasets used and/or analyzed during the current study are available from the corresponding author upon reasonable request.
